# Novel homozygous missense mutation in *GAN* associated with Charcot-Marie-Tooth disease type 2 in a large consanguineous family from Israel

**DOI:** 10.1186/s12881-016-0343-x

**Published:** 2016-11-16

**Authors:** Sharon Aharoni, Katy E. S. Barwick, Rachel Straussberg, Gaurav V. Harlalka, Yoram Nevo, Barry A. Chioza, Meriel M. McEntagart, Aviva Mimouni-Bloch, Michael Weedon, Andrew H. Crosby

**Affiliations:** 1 Department of Neurology, Schneider Children’s Medical Center of Israel, Petach Tikva, Sackler Faculty of Medicine, Tel Aviv University, Tel Aviv, Israel; 2 Medical Research, RILD Wellcome Wolfson Centre (Level 4), Royal Devon and Exeter NHS Foundation Trust, Exeter, Devon EX2 5DW UK; 3 Medical Genetics Unit, Floor 0, Jenner Wing, St. George’s University of London, Cranmer Terrace, London, SW17 0RE UK; 4 Medical Research, Diabetes group, RILD Wellcome Wolfson Centre (Level 3), Royal Devon and Exeter NHS Foundation Trust, Exeter, Devon EX2 5DW UK; 5 The Pediatric Neurology and Developmental Unit, Loewenstein Rehabilitation Hospital, Raanana, Sackler Faculty of Medicine, Tel Aviv University, Tel Aviv, Israel

**Keywords:** GAN, Charcot-Marie-Tooth disease type 2, Giant axonal neuropathy

## Abstract

**Background:**

CMT-2 is a clinically and genetically heterogeneous group of peripheral axonal neuropathies characterized by slowly progressive weakness and atrophy of distal limb muscles resulting from length-dependent motor and sensory neurodegeneration. Classical giant axonal neuropathy (GAN) is an autosomal recessively inherited progressive neurodegenerative disorder of the peripheral and central nervous systems, typically diagnosed in early childhood and resulting in death by the end of the third decade. Distinctive phenotypic features are the presence of “kinky” hair and long eyelashes. The genetic basis of the disease has been well established, with over 40 associated mutations identified in the gene *GAN*, encoding the BTB-KELCH protein gigaxonin, involved in intermediate filament regulation.

**Methods:**

An Illumina Human CytoSNP-12 array followed by whole exome sequence analysis was used to identify the disease associated gene mutation in a large consanguineous family diagnosed with Charcot-Marie-Tooth disease type 2 (CMT-2) from which all but one affected member had straight hair.

**Results:**

Here we report the identification of a novel *GAN* missense mutation underlying the CMT-2 phenotype observed in this family. Although milder forms of GAN, with and without the presence of kinky hair have been reported previously, a phenotype distinct from that was investigated in this study. All family members lacked common features of GAN, including ataxia, nystagmus, intellectual disability, seizures, and central nervous system involvement.

**Conclusions:**

Our findings broaden the spectrum of phenotypes associated with *GAN* mutations and emphasize a need to proceed with caution when providing families with diagnostic or prognostic information based on either clinical or genetic findings alone.

## Background

The classical form of giant axonal neuropathy [GAN, MIM #256850], is an autosomal recessive progressive neurodegenerative condition of the peripheral and central nervous systems [1]. A distinctive feature of the phenotype is the presence of “kinky” hair (tightly curled lacklustre hair distinctive from that of the parents) and long eyelashes. Central nervous system (CNS) involvement is variable and includes optic atrophy, nystagmus, intellectual disability and spasticity [1]. GAN is an axonal neuropathy that is typically of infantile onset and diagnosed in early childhood [2, 3] with most affected children becoming wheelchair dependent by the second decade [3, 4]. The classical form results in death in the third decade. Sural nerve biopsies taken from GAN patients, exhibit reduced density of nerve fibres and the presence of disproportionally large axons resulting from accumulation of neurofilaments [5, 6]. The causative gene was identified in 2000 [7] and over 40 different *GAN* mutations have been discovered in GAN patients with some variability in phenotype [8, 9]. Charcot-Marie-Tooth disease (CMT; also known as hereditary motor and sensory neuropathy) is a clinically and genetically heterogeneous group of peripheral neuropathies characterized by a length-dependent degeneration of motor and sensory neurons, which results in slowly progressive distal sensory loss, and weakness and atrophy of the distal limb muscles. With an estimated global prevalence of 1 in 2500, CMT is the most commonly inherited disorder of the peripheral nervous system and may show autosomal dominant, recessive and X-linked modes of inheritance. Clinically, CMT has been divided into two main subtypes; the demyelinating neuropathies (CMT-1, CMT-3, CMT-4 and CMT-X) characterized by severely reduced motor nerve conduction velocities (mNCVs) (<38 m/s), and axonal neuropathies (CMT-2) in which mNCVs are normal or only slightly reduced in association with reduced amplitude of compound motor action potentials (CMAPs). In demyelinating disease, the most frequently implicated genes in this subgroup are *PMP22*, *MPZ* and *GJB1*. In the axonal form 20% of cases are caused by mutations in *MFN2*, a dominant form, and all other forms are rare resulting from mutations in over 18 genes identified to date. It is also recognized that some genes can cause both an axonal and a demyelinating phenotype e.g. *GDAP1*. With the application of more widespread genetic testing, broadening of the phenotypic spectrum that can be seen in association with mutations in *GAN* is coming to light [10]. Recently Roth and colleagues identified two patients with biallelic mutations in *GAN*, amongst a cohort of 13 who all had straight hair [11]. These individuals had normal intellect which lead the authors to propose that the absence of “kinky” hair correlated with a lower prevalence of significant CNS involvement.

Here, we report the identification of a novel missense mutation c.103G > T; p.Val35Phe in *GAN* (NC_000016.10:g.81315216G > T; NM_022041.3:c.103G > T; p.Val35Phe), as the underlying cause of sensory-motor axonal neuropathy in a large consanguineous family apparently affected by CMT-2. Whilst affected family members all present with classical features of neuropathy including distal weakness and foot deformity, it is noted that deep tendon reflexes (DTRs) were preserved and neither nystagmus, ataxia, nor changes in white matter on magnetic resonance imaging (MRI) studies were seen. Further all but one affected member of the family had straight hair.

## Methods

### Patients and family members

**Fig. 1:**
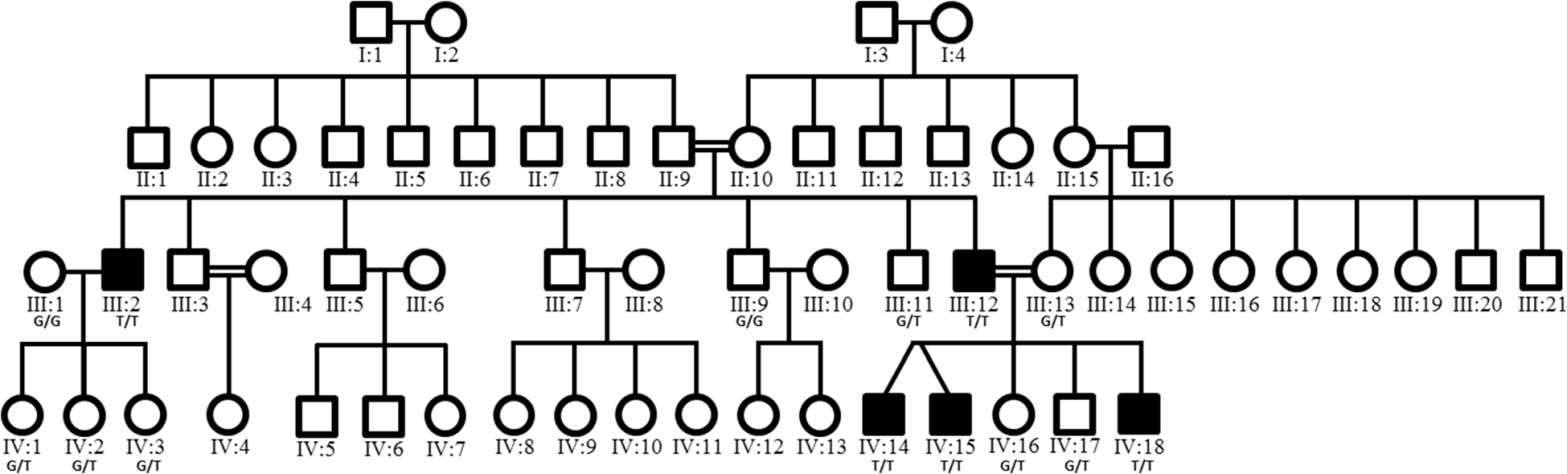
Family pedigree and c.103G > T co-segregation. Extended family pedigree of the Israeli family investigated with genotypes showing the presence or absence of GAN c.103G > T in affected and unaffected subjects respectively, who have been included in this study

This report describes genetic and clinical investigations in an extended consanguineous Muslim Arab family from a single clan residing in a small village in Israel (Fig. 1). Detailed neurological, electrophysiological, and imaging studies were performed on the patients as well as on unaffected family members.

### Molecular genetic analysis

In order to map the chromosomal location of the pathogenic variant, samples from the family were genotyped using an Illumina Human CytoSNP-12 array incorporating ~330,000 genetic markers, according to the manufacturer’s protocol.

In order to identify the disease associated gene, whole-exome sequencing was performed on a single affected individual in this family (subject III:12, Fig. 1) to generate a profile of variants not present in publically available databases and rare sequence variants. Coding regions were captured by HiSeq2000 using paired-end (2 x 100) protocol at a mean coverage depth of 30X at Otogenetics Corporation (Norcross, GA, USA). The Agilent SureSelect Human All ExonV4 (51 Mb) enrichment kit was used for exome enrichment. Sequence reads were aligned to the human genome reference sequence [hg19] and read alignment, variant calling, and annotation were performed by DNAnexus (DNAnexus Inc., Mountain View, CA; https://dnanexus.com)

## Results

### Clinical and electrophysiological findings

All clinical findings are presented in Table 1; electrophysiological findings are presented in Table 2.

**Table 1:** Summary of clinical data in affected and unaffected subjects

Individual	c.103	Sex (M/F)	Onset	Distal weakness	DTR	Kinky hair	Foot deformity	Nystagmus	MRI white matter changes	Ataxia	Other features
Affected Individuals											
III:2*	T/T	M	1^st^ decade	+++	- UE	-	Yes	No	N/A	No	Facial weakness. Speech difficulties
					+ pat						
					- acill						
III:12	T/T	M	1^st^ decade	+++	+	-	Yes	No	N/A	No	
IV:14	T/T	M	6y	++	+	-	Yes	No	No	No	
IV:15	T/T	M	6y	++	+	-	Yes	No	No	No	
IV:18	T/T	M	4y	+	++	curly	Yes	No	No	No	
Unaffected Individuals											
III:1	G/G	F	-	-	++	-	No	No	N/A	No	
III:9	G/G	M	-	-	++	-	No	No	N/A	No	
III:11	G/T	M	-		++	-	No	No	N/A	No	
III:13	G/T	F	-	-	++	-	No	No	N/A	No	
IV:1	G/T	F	-	-	++	-	No	No	N/A	No	
IV:2	G/T	F	-	-	++	-	No	No	N/A	No	
IV:3	G/T	F	-	-	++	-	No	No	N/A	No	
IV:16	G/T	F	-	-	++	-	No	No	N/A	No	
IV:17	G/T	M	-	-	++	-	No	No	N/A	No	

*Individual III:2 is the father of individuals IV:14, IV:15, and IV:18 (see Fig. 1)

*DTR* deep tendon reflexes, *UE* upper extremities, *pat* patella, *acill* Achilles, *N/A* not available

**Table 2:** Electrophysiological data

Individual	Age (years)	Latency (ms)	CMAP (mV)	Motor nerve velocity (m/s)	Sensory nerve latency (ms)	SNAP (μV)
II:9	70	Median-4.6 (*n* < 4.2)	Median-13.9 (*n* > 4)	Median- 52 (*n* >49)	Median- 3.8 (*n* < 2.5)	Median-2.4
		Ulnar- 3.7 (n)	Ulnar-8.3 (*n* > 6)	Ulnar-52 (n)	Ulnar-3.5 (*n* < 2.1)	
		Peroneal- 5.9 (*n* < 5.2)	Peroneal-1.7 (*n* > 2)	Peroneal-33 (*n* > 40)	Sural-absent	Sural- absent
II:10	70	Median 10.8	Median-1.5	Median- 38	Median – N/A	Median-absent
		Ulnar-3.6 (n)	Ulnar-10	Ulnar-.56 (n)	Ulnar-2	Ulnar-6.2
		Peroneal-5.1	Peroneal- 1	Tibial- 42	Sural-2.9 (*n* < 3.2)	Sural-19
III:12*	33	Median-5.5	Median-5.4	Median- 55.6	absent	absent
		Ulnar- 5.6	Ulnar-1.5	Ulnar-50		
		Tibial& peroneal- N/A	Tibial-absent	Tibial-absent		
			Peroneal- absent	Peroneal- absent		
IV:14*	6	Median-3.1	Median-6.1	Median- 60.7	Median- 2.8	Median-22
		Ulnar-2.8	Ulnar-5	Ulnar-60	Ulnar-2.4	Ulnar-25
		Tibial-4.2	Tibial-1.3	Tibial-35.6		
		Peroneal-4.1		Peroneal-56.4		
		normal				
IV:15*	6	Median-3.7	Median-5.7	Median- 51.7	Median- 3	Median-56
		Ulnar- 2.9	Ulnar-6.3	Ulnar- 66	Ulnar-3	Ulnar-42
		Tibialis-5.1	Tibialis-3.1	Tibial- 41.8	Sural-3.5	Sural-9.9
		Peroneal-4.1	Peroneal-0.5	Peroneal- 47.7		
IV:18*	8	Median-4.5	Median-3.6	Median-64	absent	absent
		Tibialis-4.4	Tibialis-1.4	Tibialis-50		
		Peroneal-5.7	Peroneal-2.1	Peroneal-59		

*Affected individual

*N/A* not available

#### Index patients: IV:14 and IV:15

The index patients (IV:14, IV:15) are dizygotic twins, born to consanguineous parents (first cousins) after an uneventful pregnancy and delivery. Gross motor development was slow (both walked at age 30 months), but fine motor and speech development was normal. Both attended regular school, and academic performance was satisfactory.

The patients initially presented at our clinic at age 6 years for evaluation of abnormal gait. On physical examination, no dysmorphic features were noted. Hair was normal and not kinky. There were no cranial nerve abnormalities and no signs of nystagmus. Weakness of the ankle and toe extensors and flexors, peroneus muscles, and tibialis posterior muscles was documented (Medical Research Council grade 4/5), with slightly decreased vibration. DTRs were elicited. Pes planus was noted. Both patients exhibited a crouched gait pattern with externally rotated feet. Cerebellar function was normal. Neurophysiological studies revealed normal motor and sensory nerve conduction velocities, in association with a reduced amplitude CMAP of the right tibial nerve in patient IV:14 and of the right peroneal nerve in patient IV:15. MRI of the brain did not reveal white matter changes.

Tendon-lengthening surgery was performed to treat the contractures. The most recent follow-up evaluation was conducted at age 15 years. The distal weakness was more pronounced, although the patients were still ambulatory. One of the twins was overweight, further exacerbating his walking difficulties.

#### Patient III:12 – Father of the index patients

The father of the index patients (patient III:12), whose parents were also consanguineous, had been diagnosed with Charcot-Marie-Tooth and was wheelchair-bound by age 12 years. He completed 12 years of school despite mild learning difficulties.

Examination at the time of admission of the index patients, at age 33 years, revealed no dysmorphic features or hair abnormalities. Findings on cranial nerve examination were normal. Atrophy of the upper limbs was documented, with weakness of the small hand muscles, mainly interosseous. Vibration was slightly reduced, but sensation was otherwise normal. The lower limbs exhibited normal proximal muscle strength but severe weakness of the ankle and toe flexors and extensors, in addition to reduced vibration and proprioception of the toes. Deep tendon reflexes were elicited. Neurophysiology studies demonstrated widespread severe, predominantly axonal, sensorimotor neuropathy more marked in the legs.

#### Patient IV:18 – Brother of the index patients

The brother of the index patients (IV:18) presented to our clinic at age 4 years because of maternal concerns about gross motor impairment, similar to findings in his older brothers. He fell frequently. Past medical history revealed an uneventful pregnancy and delivery. Walking started at age 18 months and speech was delayed. He had moderately curly hair but it was not neither kinky nor frizzy. Other than curly hair, there were no dysmorphic features. Distal weakness was noted in the lower limbs with preserved reflexes, in addition to pes planus, crouched gait, and externally rotated feet. Babinski sign was positive. Neurophysiology studies performed at age 8 years demonstrated a mild axonal motor sensory polyneuropathy in the legs and arms with a mild-moderate reduction in compound motor action potential amplitude and absence of sensory conduction. Needle electromyography revealed no abnormalities except for mild early chronic reinnervation in the right tibialis anterior muscle. MRI of the brain was normal, with no apparent white matter changes.

#### Patient III:2 – Paternal uncle of the index patients

The index patients’ paternal uncle. He had frequent falls in childhood and later complained of hand weakness. He was wheelchair-bound in his mid-twenties. Although he was not diagnosed with learning difficulties, he did not proceed to high school. Examination at age 39 years, a few years after our index patients' admission, revealed no dysmorphic features or hair abnormalities. Mild facial weakness and speech dysarthria was noted. Atrophy of the upper limbs was documented with weakness of the small hand muscles, mainly interosseous. The lower limbs exhibited mild proximal muscle weakness, severe distal weakness, in addition to reduced sensation vibration and proprioception of the toes. DTRs were not elicited in the upper limbs. Neither were they elicited in the left patella and both Achilles tendons, however this is attributed to tendon-lengthening surgery. Foot deformity was noted.

### Genetic findings

**Fig. 2:**
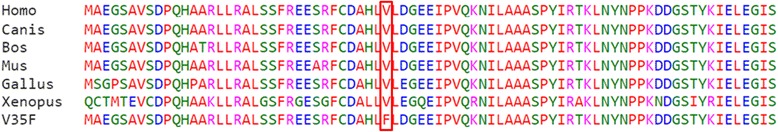
Protein homology of GAN in various species and the p.Val35Phe mutant molecule. The mutation results in an amino acid substitution at Valine (indicated by the red box) to phenylalanine at position 35, a residue highly conserved across species

Inspection of the SNP genotypes of affected family members identified a single notable homozygous region of 2.15 Mb on chromosome 16q23.2-23.3, delimited by markers rs889518 and rs4783311 (Chr16:80,897,297-83,051,118 [hg38]), shared solely by affected individuals in the family. Multipoint linkage analysis was performed with Simwalk2 [12], using the SNP genotype data, assuming autosomal recessive inheritance, full penetrance and a disease allele frequency of 0.00001, achieving a LOD_MAX_ = 2.71 corresponding to the autozygous region. This region contains 31 genes and was considered very likely to harbor the pathogenic variant. After filtering, exome sequencing of DNA from a single affected individual identified only one likely deleterious variant within the 16q23.2-23.3 region, a single base substitution (NC_000016.10:g.81315216G > T; NM_022041.3:c.103G > T; p.Val35Phe) in *GAN*. Dideoxy sequence analysis confirmed co-segregation of the variant with the disease phenotype in the family with unaffected family members being wildtype or heterozygous for the variant. The variant is not present in genomic databases (Exome Variant Server, EVS; dbSNP; Human Gene Mutation Database, HGMD; ExAC). The c.103G > T nucleotide alteration is predicted to affect a stringently conserved amino acid across all species examined, and substitute a valine at codon 35 to phenylalanine (p.Val35Phe, Fig. 2). *In silico* mutation analysis provided conflicting predictions as to the functional outcome of the variant on the gigaxonin protein: PolyPhen-2 [13] HumDiv and HumVar both predicted the variant to be benign with scores of 0.108 and 0.021 respectively, while SIFT and PROVEAN [14] predicted the variant to be disease causing with scores of 0.017 and −2.78, respectively.

Unaffected family members had either a homozygous normal (G/G) phenotype or heterozygous G/T genotype, consistent with the recessive mode of inheritance of GAN. None presented any distal weakness, foot deformity, ataxia or nystagmus.

## Discussion

In this work, a novel sequence alteration in the gene *GAN*, c.103G > T, was identified as most likely the underlying cause for a sensory-motor axonal neuropathy in a large consanguineous family presenting as CMT-2. Affected family members were all male, homozygous for the mutation and all presented with distal weakness, more pronounced in older individuals (generation III), consistent with the progressive nature of GAN. They all had foot deformities but none had nystagmus or ataxia and all had preserved DTR. Findings in clinical and imaging analyses, as well as medical history did not suggest CNS involvement. There was no evidence of significant intellectual disability or seizures. MRI studies, available only for the two index patients and their brother revealed no white matter changes. Nerve conduction studies showed reduced amplitudes with prolonged distal latencies and normal or prolonged conduction velocities which is consistent with axonal motor-sensory neuropathy.

The clinical presentation of affected individuals in this current family differs from the consanguineous Arab-Israeli family with GAN described by Abu Rashid and colleagues [8]. Although an intermediate phenotype, their patients had kinky hair, thick eyelashes and thick eyebrows, ataxia, spasticity and MRI changes, all characteristic of GAN and CNS involvement.

The gene *GAN* encodes the protein gigaxonin, a BTB-KELCH protein that plays a role in regulating intermediate filaments (IFs) [15] and the degradation of MAP1B light chain, which is critical to neuronal survival [16]. Based on sequence homology, it is predicted to be a substrate adaptor for Cul3-E3 ubiquitin ligase, and hypothesized to be involved in the ubiquitination and degradation of several IF proteins, including vimentin [17].

The c.103G > T nucleotide change is predicted to affect a stringently conserved amino acid across all species examined, and substitute a valine at residue 35 to phenylalanine (p.Val35Phe) within the BTB-domain of the protein. The BTB-domain has been established as the proteins homodimerization domain [18, 19], as well as its site of interaction with the E3 ligase, MYCBP2, and the heat shock proteins, HSP90AA1 and HSP90AB1 [20]. Boizot and colleagues showed that missense mutations in this region (p.Arg15Ser and p.Ala49Glu) can lead to destabilization of the protein resulting in a shorter half-life and lowered protein abundance [19]. Their predictions support the *in-silico* prediction of SIFT and PROVEAN [14] that our p.Val35Phe variant is disease-causing. Of note, they describe the phenotype of the p.Arg15Ser mutation as a moderate form of GAN, somewhat similar to ours, with very slow progression, no central nervous impairment and extended survival. Roth and colleagues [11] conducted a GAN natural history study on 13 patients, two of whom (c.130C > T, p.Gln44X; c.1420G > C, p.Gly474Arg; c.971C > T, p.Ala324Val; c.1391G > A, p.Cys464Tyr) lacked the characteristic tightly curly (kinky) hair. These patients’ gross motor function measure was better than those with curly hair in the cohort and defined their phenotype as milder. Both straight-haired patients in the Roth study had a distinctly different phenotype from ours (including occolomotor apraxia as compared to complete absence of ocular signs in our patients).

Zemmouri and colleagues [21] have described a consanguineous Algerian family with a CMT2-like presentation of GAN, characterized by late onset and little to no involvement of CNS. They were able to establish a linkage to the chromosome 16q locus, however, they did not find the exact nature of the mutation which was reported by Bormont and colleagues the same year [7].

In this study we found a novel missense mutation in the gene *GAN* in a consanguineous family that is most likely the underlying cause of a CMT2-like presentation of GAN. In the post- genomics era with easy access to gene panels and exome sequencing for clinical investigation the family presented here serves to highlight the need to proceed with caution when giving out prognostic information based on the genetic findings. The spectrum of phenotypes associated with mutations in many genes is broadening due to our ability to identify these variants with whole exome/genome sequencing in the absence of the classical phenotype. Classical GAN is a devastating disease but the family described here and reports of families such as documented by Roth and Abu-Rashid emphasize the need to acknowledge a wider phenotypic spectrum when counseling such families.

Further research is needed to elucidate the molecular mechanisms mediating the effects of mutations in specific Gigaxonin protein domains on aspects of the GAN disease presentation.

## Conclusion

Our findings broaden the spectrum of phenotypes associated with *GAN* mutations and emphasize a need to proceed with caution when providing families with diagnostic or prognostic information based on either clinical or genetic findings alone.

## Abbreviations

Ala: (amino acid symbol) Alanine
Arg: (amino acid symbol) Arginine
CMAPs: Compound motor action potentials
CNS: Central nervous system
Cys: (amino acid symbol) Cysteine
DTRs: Deep tendon reflexes
EMG: Electromyography
F: Female
*GAN*: (gene symbol) Giant axonal neuropathy
GAN: Giant axonal neuropathy
*GDAP1*: (gene symbol) Ganglioside induced differentiation associated protein 1
*GJB1*: (gene symbol) Gap junction protein, beta 1
Gln: (amino acid symbol) Glutamine
Gly: (amino acid symbol) Glycine
HSP90AA1: (protein symbol) Heat Shock Protein 90 kDa Alpha (Cytosolic), Class A Member 1
HSP90AB1: (protein symbol) Heat Shock Protein 90 kDa Alpha (Cytosolic), Class B Member 1
Ifs: Intermediate filaments
M: Male
m/s: Meters per second
*MFN2*: (gene symbol) Mitofusin 2
mNCVs: Motor nerve conduction velocities
*MPZ*: (gene symbol) Myelin protein zero
MRI: Magnetic resonance imaging
ms: Milliseconds
mV: Millivolts
MYCBP2: (protein symbol) MYC Binding Protein 2, E3 Ubiquitin Protein Ligase
Phe: (amino acid symbol) Phenylalanine
*PMP22*: (gene symbol) Peripheral myelin protein 22
SNAP: Sensory nerve action potential
Tyr: (amino acid symbol) Tyrosine
Val: (amino acid symbol) Valine
μV: Microvolts
